# Analysis of the Influence of Cracked Sleepers under Static Loading on Ballasted Railway Tracks

**DOI:** 10.1155/2014/363547

**Published:** 2014-10-28

**Authors:** Laura Montalbán Domingo, Clara Zamorano Martín, Cristina Palenzuela Avilés, Julia I. Real Herráiz

**Affiliations:** ^1^Institute for Multidisciplinary Mathematics, Polytechnic University of Valencia, Camino de Vera s/n, 46022 Valencia, Spain; ^2^Foundation for the Research and Engineering in Railways, 160 Serrano, 28002 Madrid, Spain

## Abstract

The principal causes of cracking in prestressed concrete sleepers are the dynamic loads induced by track irregularities and imperfections in the wheel-rail contact and the in-phase and out-of-phase track resonances. The most affected points are the mid-span and rail-seat sections of the sleepers. Central and rail-seat crack detection require visual inspections, as legislation establishes, and involve sleepers' renewal even though European Normative considers that thicknesses up to 0.5 mm do not imply an inadequate behaviour of the sleepers. For a better understanding of the phenomenon, the finite element method constitutes a useful tool to assess the effects of cracking from the point of view of structural behaviour in railway track structures. This paper intends to study how the cracks at central or rail-seat section in prestressed concrete sleepers influence the track behaviour under static loading. The track model considers three different sleeper models: uncracked, cracked at central section, and cracked at rail-seat section. These models were calibrated and validated using the frequencies of vibration of the first three bending modes obtained from an experimental modal analysis. The results show the insignificant influence of the central cracks and the notable effects of the rail-seat cracks regarding deflections and stresses.

## 1. Introduction

Railway tracks consist of several components grouped into two categories: substructure and superstructure. The substructure includes ballast, subballast and subgrade while the superstructure includes sleepers, rail pads, fasteners and rails. Sleepers are the track components of ballasted track which rest on the ballast transversely, provide support and fixation to the rails, and transmit the stresses to the granular layers. The majority of modern railway sleepers used worldwide are prestressed concrete sleepers.

The loading conditions acting on railway tracks are normally time dependent since the wheels, moving at the train speed, interact with rails. As a result, not only static or quasistatic loads appear in the track, but also dynamic loads. The dynamic loads are frequently caused by the track irregularities, irregular track stiffness due to variable properties, and settlement of ballast bed and formation (unsupported sleepers); rail corrugation; wheel flats and shells; worn wheels and rail profiles and discontinuities at welding points, joints, and switches; hunting or resonance vibrations [1].

The impact loads, which are part of the dynamic loads, are infrequent and of short duration but high magnitude. The typical magnitude of these impact loads (wheel/rail forces) from the reviewed cases in heavy haul traffic by Remennikov and Kaewunruen [2] varies roughly between 100 kN up to 750 kN, depending on the causes and the speed of the train.

The principal causes of cracking in prestressed concrete sleepers are the underdimensioning and/or the underestimation of the actions on the track. These impact loads are mainly the cause of increase of the forces on the track that finally cause cracking in the sleepers [3]. Moreover, it was found that the in-phase and out-of-phase track resonances in old and bad-conditioned tracks are likely to associate with the first bending and second bending modes of vibration of the sleepers, respectively. This confirms the knowledge that at certain wheel loading frequencies the sleepers tend to dramatically vibrate and develop cracks at the bottom of rail-seat or at the top surface of mid-span [4]. Esveld [1] discovered that the ballast breakage increases substantially track resonance, so-called in-phase vibration. This phenomenon causes voids and pockets, or even the poor compaction of the ballast support underneath the railway concrete sleepers [5, 6]. These voids and pockets would also allow the sleepers to vibrate freely with greater amplitudes and lead to larger crack widths or fatigue fracture [6].   Moreover, the dynamic loads often excite the railway track components with increased magnitudes at specific frequencies associated with such components. It was found that the railway concrete sleepers deteriorate greatly when they are subjected to dynamic loads at their resonant frequencies, especially in flexural modes of vibration [2, 5]. These studies also showed that the interaction between the sleeper and the underlying ballast can be of importance for the dynamic behaviour of the sleeper. During a train passage, the time histories of the vertical displacement for the sleeper and the ballast can involve oscillation out-of-phase. This results in large impact forces since the sleeper hits the ballast surface [7].

Taking into account these investigations, it is clear that the most loaded sections in the sleepers are two: the mid-span and the rail-seat section. The central section presents the maximum bending moment, which in case of bad maintenance may be increased due to a tamping lack, voids or pockets, or track resonance. On the other hand, the rail-seat section receives a big percentage of the dynamic loads produced in the wheel-rail contact depending on the characteristics of the fastenings and pads and is also affected by the track resonance, which may aggravate the cracking.

Regarding the concept of track maintenance, it is defined as the total process of maintenance and renewal required to ensure that the track meets safety and quality standards at minimum cost. The renewal is necessary in cases where the needs of maintenance activities are excessive. With reference to the sleeper renewal, the present Spanish legislation establishes that frequent visual inspections must be performed to check the appearance of cracks and indicates that those damaged sleepers with cracks that may affect the track must be renewed. However, cracks generated in the bottom of the rail-seat section are not visible because of the ballast. Moreover, the legislation does not establish a criterion to distinguish when a single crack is affecting the track or the manner in which it influences the infrastructure.

Concerning this situation, it is a matter of utmost interest to analyse the influence of the cracked sleepers on the track global response in terms of deflections during the vehicles passage as well as the stresses registered in the different layers that constitute the track and the variation of the vertical track stiffness. Although more parameters should be considered to make a decision about the need of replacing a sleeper, this research intends to evaluate their influence only from a structural point of view, considering the global railway track structure.

The Finite Element Method constitutes a very useful tool to study the railway track structure. It was firstly used in parametric studies by Desai et al. [8], Profillidis [9], or Shahu and Rao [10], for example. In particular, the studies developed by the E.N.C.P. (École Nationale des Ponts et Chausées) in the 1980s using the program ROSALIE [9] were integrated in the D-117 ORE technical committee, which validated the results obtained with extensive experimental measurement campaigns in France, Great Britain, and Austria. These results were the base for designing abacus of the supporting structures developed by the UIC in the present structural sections catalogue [11]. It has been used in the design of railway track substructure [12, 13]; in the prediction of stresses and displacements inside the track support structure [14]; in the study of the influence of design parameters on dynamic response of the railway track structure [15]; in the design [16] and effects [17] of embankment-structure transitions [16]; and in researches about dynamic response of track-embankment ground system influenced by train moving loads [18]. Additionally, Finite Element Method has been used for crack analysis. Cracks are frequently found in many engineering structures, such as rock and concrete, and such cracks usually play a determinant role in the structural stability [19]. One example of the use of this method in civil engineering structures is the study about the effects of outlets on cracking risk and integral stability of super-high arch dams carried out in [20]. In railroad researches, Finite Element Method has also been used to study cracks. For instance, wheel wear and rolling contact fatigue crack initiation have been modelled using the Finite Element Method [21].

With regard to previous works related to the issue, Gustavson and Gylltoft [7] developed a global track model together with a Finite Element Model of the sleeper to analyse the influence of cracks in sleepers on the response of the entire track during a train passage. The sleeper model simulated the cracking phenomena at the rail-seat due to rail corrugation for train passages at 130 km/h and its effect was introduced on the separate track model by enforcing the displacement history to connect both models. Results showed that reducing Young's modulus of the concrete in the linear analysis by about 10 percent produced results similar to those from the nonlinear analysis. The simulations with the track model led to the conclusion that decreasing flexural stiffness of the sleepers by 10 percent, (i.e., considering the crack) had minor influence on the direct global response of the track. This research made an important study about cracked sleepers under rail-seat section. Nevertheless, this study did not discuss the stresses on the track and the effect of more than one cracked sleeper which is the common real situation in tracks.

The concrete sleepers European Normative establishes that an adequate behaviour is expected if crack openings remain lower than 0.5 mm; nevertheless, the Spanish Normative determines as mandatory the renovation of those cracked sleepers that may alter the correct operation and the safety of the track. From this point of view, this paper intends to study how the cracks at the central or rail-seat section influence the track response under static loading for different crack thicknesses. The track model considers three different sleeper models: uncracked, cracked at central section, and cracked at rail-seat section. These models were calibrated and validated using the frequencies of vibration for the first three bending modes obtained in experimental modal analysis. The track model with uncracked sleepers was calibrated and validated using vertical deflections measured in a real track.

## 2. Cracked Sleeper Model

From previous works [12, 16], it is deduced that it is possible to model a sleeper as a prismatic element with constant section *A* and length *L*, with moment of inertia *I* and constituted by an uniform material with density *ρ* and equivalent modulus of elasticity *E*. The use of an equivalent modulus of elasticity that includes the behaviour of the concrete and the prestressing tendons is a validated technique to simplify the model [12]. In order to improve the accuracy of the model and considering that the cross section of concrete sleepers is not constant; the sleeper was divided into elements with constant cross sections. To maintain the flexural stiffness of each division, the product of the inertia and the modulus of elasticity (*E*_*I*_) of each sleeper element must be equal in the sleeper element modelled and in the real one (1).

(1) $$\begin{array}{c} {\left(E_{I}\right)}_{\text{model}}={\left(E_{I}\right)}_{\text{real}}. \end{array}$$

Taking into account that the moment of inertia in the model is constant because the cross section is uniform, different modulus of elasticity was considered along the sleeper model to satisfy the equality between the flexural stiffness of each element. The modified modulus of elasticity in each section was calculated using (2):

(2) $$\begin{array}{c} E_{\text{model}}=\frac{E_{\text{real}}\cdot I_{\text{real}}}{I_{\text{model}}}. \end{array}$$

**Figure 1:**
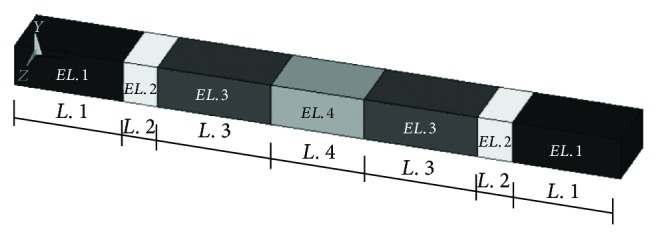
FE model of the uncracked sleeper.

The section geometry of the model is defined by the rail-seat section. The width of the model, *B*, corresponds to that of the real sleeper at this section so that the loaded area in static conditions remains the same. Therefore, the model section height, *H*, was calculated taking into account that the moment of inertia must coincide with the real one.  Figure 1 shows the model geometry and the elements distribution.

The Young's modulus resulted to be the only variable to be calibrated in order to reproduce the exact behaviour of the sleeper. The calibrated Young's modulus corresponds to the rail-seat block; the rest may be deduced using (1). With respect to Poisson's ratio, a value of 0.25 is recommended for monoblock concrete sleepers [12].

The model updating was performed using the three first frequencies of the bending modes of vibration in the vertical plane, which were obtained by the application of an experimental modal analysis to uncracked sleepers. All the calculations of the model have been performed using the Modal analysis provided by ANSYS v11.0., which solves (3):

(3) $$\begin{array}{c} \left[M\right]\left\{\ddot{u}\right\}+\left[K\right]\left\{u\right\}=\left\{0\right\}, \end{array}$$

where *M* is the mass matrix, *K* is the stiffness matrix, *u* is the displacement vector, and $\ddot{u}$ is the acceleration vector.

The properties of the uncracked model are listed in Table 1.

**Figure 2:**
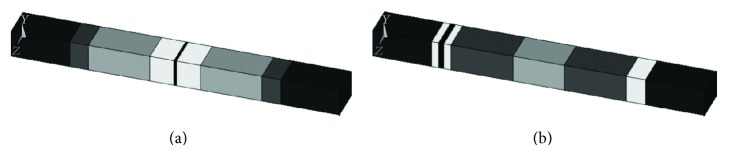
FE of the sleeper cracked at its central section (a) and at rail-seat section (b).

Once the model of the uncracked sleeper was correctly developed, it was necessary to introduce in the model some modifications in order to simulate the effect of the crack. A crack is a discontinuity in the material that introduces a decrease in the cross section inertia since less part of the concrete section is working. Thus, the cracks weaken the bearing capacity of the sleeper [22]. This decrease in the inertia has associated a decrease in the flexural stiffness. Since the inertia could not be modified because of the geometric characteristics of the model, the same effect was achieved by introducing a decrease of the Young's modulus in the area where the crack appears. That is, the crack was simulated as a volume of more flexible material as observed in Figure 2.

The calibration of the model [23] was also performed using the results of an experimental modal analysis applied to cracked sleepers and it provided the characteristics of the flexible zone: the thickness and Young's modulus of the cracks at the central section and the rail-seat section led to the results in Table 2.

The good correlation between the three first resonant frequencies of the vertical bending modes of the real sleepers and the numerical models ensured the adequate simulation of the structural behaviour.

## 3. Track Model

The track model used in this study was designed based on the recommendations provided by Ministerio de Fomento [12] and Gallego [24], exception made of the sleeper model and the meshing. The models to be considered are the cracked and uncracked sleeper models detailed in the previous paragraphs and the meshing was adapted to the modifications introduced by these new models.

The calibration and validation of the track without cracks model (uncracked track model) were done following the procedure explained afterwards. In this point, it is important to consider that uncracked track model characteristics have been calibrated using real vertical deflections of the rail top head and validated using real vertical deflections of the different elements over the track platform considering uncracked sleepers.

Firstly, a sensitivity analysis was carried out to obtain the significant variables in the calibration process. Embankment height, embankment Young's modulus, pad Young's modulus, and subballast height were the variables considered in this analysis. The value ranges of these variables were the commonly used in Spanish railway networks [12]. The sensitivity analysis showed that all these variables affected the vertical displacement of the rail top head values; therefore all these variables were considered as significant. Secondly, these variables were used for the uncracked track model calibration. A desirability function implemented in Statgraphics software package was used to obtain the objective variables values among 81 possible combinations studied. Finally, the calibration of the model was obtained with this function as indicated in Table 3.

Thirdly, the model validation was performed to ensure the proper track model functioning. The results obtained in this part were satisfactory as shown in Table 4.

The values of the variables considered, which have been calibrated and validated, can be observed in track model description, which is made in the following sections.

Once the uncracked track model was calibrated and validated, it was possible to couple the calibrated model of the cracked sleeper obtaining the cracked track model [7]. With this procedure, different track models (uncracked and cracked) were implemented to obtain the necessary results for this study.

### 3.1. Problem Domain

**Figure 3:**
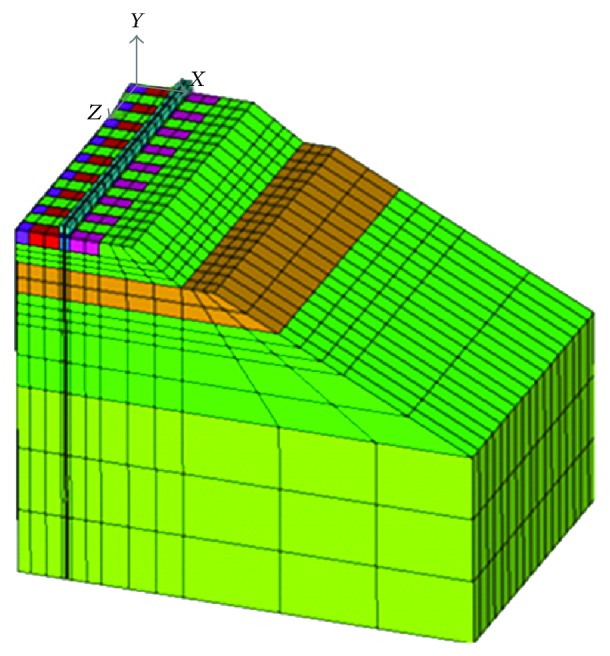
Track model axis.

The geometry of the problem was considered in order to correctly simulate the mechanical response. In the model, the *x*-axis is the cross direction, the *y*-axis is the vertical direction, and the *z*-axis is the longitudinal direction. The cross section is composed of superstructure and substructure elements. The track was modelled as a single track symmetrical about the plane *x* = 0 so as to reduce the computational time. The geometry of the analysed track is symmetric about the *x*-axis (*y*-*z* plane) and the *z*-axis (*x*-*y* plane). No simplifications were made regarding the *z*-axis to avoid the influence of the boundary conditions on the results.  Figure 3 shows the track model and the axis used.

In the longitudinal direction, a length equivalent to nine sleepers with 60 cm of distance between them was analysed. The reason lies in the influence of an applied load at any point of the track: according to the ORE Committee D-117, it has been proven to be transmitted to the adjacent sleepers, decreasing until approximately the fourth sleeper from the loaded sleeper. Taking into account four sleepers in each direction and the loaded sleeper, the total length of the model is 5.07 m.

### 3.2. Geometry and Element Discretization

The substructure elements were modelled using methods proposed in other studies [12, 24].

**Figure 4:**
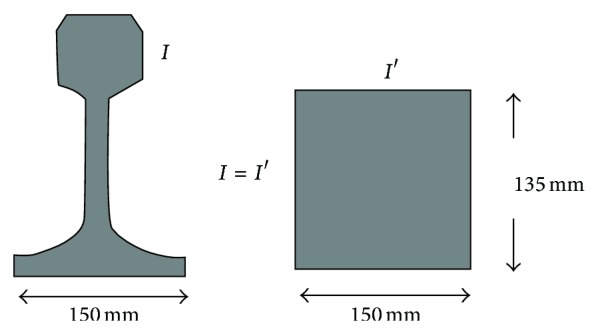
Rail modellization.

The rail is the UIC 60. To model it, an equivalent rail of the same material but with a rectangular section was assumed. The width of the rectangle is equal to the width of the rail foot and the height is calculated to equal the actual rail stiffness, according to Figure 4.

The concrete sleepers were introduced in the track model as explained before, in the cracked and uncracked case. The rail pads were modelled with new dimensions to be compatible with the rails and sleepers geometry and ensuring that the vertical stiffness was equivalent to the vertical stiffness provided by the manufacturer. The rail pad surface had to be equal to the contact surface between the rail and sleeper. Hence, the thickness (*h*) was calculated as shown in (4):

(4) $$\begin{array}{c} E=k\cdot \frac{h}{S}. \end{array}$$

The ballast, subballast, blanket, embankment, and foundation did not require any simplification as they are defined by their thicknesses, which are 0.3 m, 0.5 m, 0.5 m, 1 m, and 3 m, respectively, and their slopes are 3 : 2, 2 : 1, 2 : 1, 2 : 1, and 0 : 1. To mesh the model, 20-node brick-type elements were used as they were able to reproduce flexion mechanism, a significant resistance mechanism for the track grid (rail and sleepers) [12].

### 3.3. Boundary Conditions

The boundary conditions in the three directions were defined as follows. The surfaces of the embankment slopes are completely free. The sections bordering the model in the longitudinal direction have restricted displacements at the *z*-axis. The sections bordering the model in the cross direction have restricted displacements at the *x*-axis. Finally, the horizontal surface at the bottom of the model has restricted vertical displacements.

In order to link the node displacements of the adjacent nodes, the evolution of the displacements was considered to be continuous. Special consideration is required for sleeper-ballast contact modelling, which involves independent movements of the nodes within the interface. The most common solution to modelling the contact zones is to use bounded degrees of freedom. In fact, this solution was adopted by ORE Committee D-117 and was used by the Railway Track Formations Project in its recommendations on railway track construction [12]. The use of bounded degrees of freedom requires the introduction of different nodes for each material at the contact surface. These nodes must move equivalently in the direction perpendicular to the contact plane. However, these nodes can move at different values in the directions parallel to the contact plane. Additionally, the bond between granular materials is adherent and they share the nodes at the contact surface.

### 3.4. Material Constitutive Models

The mechanical behaviour of a railway structure is determined by the displacements, strains, stresses, and external loads. These variables are related to each other by equilibrium equations, kinematic equations, and constitutive equations. The constitutive equations of the materials must represent the real response of each material.

Sleepers, rails, and rail pads were introduced into the model as elastic linear materials for the range of stresses studied. This simplification cannot be adopted for granular materials (foundation, embankment, blanket, ballast, and subballast). Although these materials have an elastic response under very low levels of stress, they exhibit a plastic behaviour under higher stresses, which occurs in our case [16]. For this reason, elastoplastic behaviour was assumed. This assumption implies that reloading occurs in the same manner as unloading; therefore, the material experiences no hardening [16]. The most appropriate model to represent this phenomenon is the Drucker-Prager model [25] which is based on the hydrostatic stress. This model was applied to this study because it was used in several railway design studies and has been validated by the ORE Committee D-171. It shows that an elastoplastic response of the granular material is suitable for the modelling. This model of behaviour may be introduced in ANSYS by implementing the cohesion, *c*, and the angles of internal friction (*Ф*_*i*_) and dilatancy (*Ф*_*d*_) of each material. The properties of the elements that constitute the railway track are summarized in the Table 5.

Due to the nonelastic nature of granular materials, the strains of the structure depend on the load history [16].

To assess the effects produced by trains, loading was conducted in two stages. In the first stage, only the material's own weight was considered. In the following stage, the load due to the train passage was also taken into account. Stresses and displacements of interest are the ones that correspond to the application of the train loads; therefore, they can be calculated from the difference between the totals obtained after applying the train loads to the first stage.

The loads of the train were introduced as vertical loads applied at the central point of the rail and represent tons per wheel transmitted by the train to the track. The vehicle static load differs from the actual load transmission due to dynamic forces that appear in the wheel-rail interface as a result of vertical movement. This vertical movement is a consequence of the system alteration because of track and wheel defects. The analysis is static, with an amplified load to account for the dynamic effects. The ballast and subballast thicknesses are considered to be adequate to ensure correct behaviour under cyclic loads [26]. Thus, the failure modes are not studied. In this model, the value of the increased dynamic load was calculated using Eisenmann's formula [27] in (5):

(5) $$\begin{array}{c} Q_{d}=Q_{e}\cdot \left[1+t\cdot \overset{-}{s}\cdot \left(1+\frac{V-60}{380}\right)\right]. \end{array}$$

This investigation presents the results obtained for a high-speed line. The load considered in the analysis corresponds to passenger vehicles (17 t/axis). Specifically, speed of 300 km/h was used. In the calculations, the statistical security coefficient (*t*) is 2, corresponding to the percentile of 95.9% and *s* is 0.2, regarding the good conditions of the track. The result is 280.9 kN/axis, so a load of 140.45 kN/wheel was considered in the calculations.

## 4. Results

The results shown correspond to each of the case studies: railway track with uncracked sleepers, with 1 to 6 cracked sleepers at the central section, and 1 to 6 at the rail-seat section and are presented here so as to establish a comparison between them and study the influence of the cracks.

The first situation to be analysed is the uncracked case in order to obtain the reference situation to compare the following cases. The cases with cracked sleepers were divided into cracks located at rail-seat section or at central section. Each one of these cases took into account the corresponding cracked sleeper model. It is known by experience that cracking occurs in groups of sleepers, not in an individual way, so different combinations were considered. The most significant stresses in the stress state of each element are the vertical stresses: they are caused by the passing of vehicles and are transmitted from the wheel-rail contact to the foundation through each element of the structure. The points where the deflections and stresses have been calculated correspond to those in a limit situation in the vertical line under the load application point. According to this, the results were obtained in the rail head, the upper part of the sleeper, and the contact interfaces between sleeper-ballast, ballast-subballast, subballast-blanket, and blanket-embankment. The results are presented in the following in Tables 6, 7, 8, and 9.

**Figure 5:**
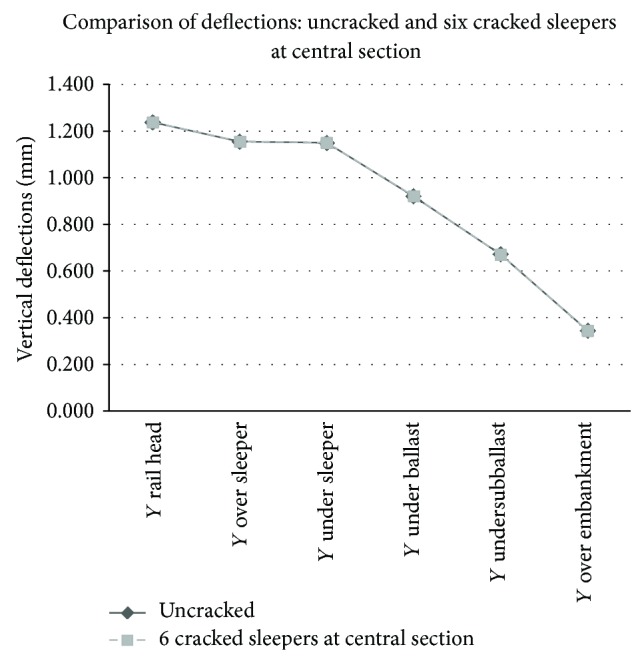
Comparison of deflections (mm) in a track with healthy sleepers and six cracked sleepers at central section.

**Figure 6:**
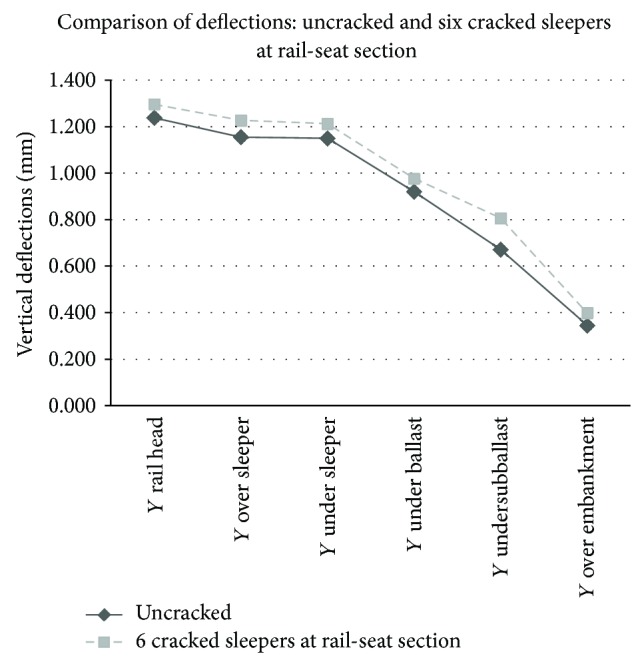
Comparison of deflections (mm) in a track with healthy sleepers and six cracked sleepers at rail-seat section.

**Figure 7:**
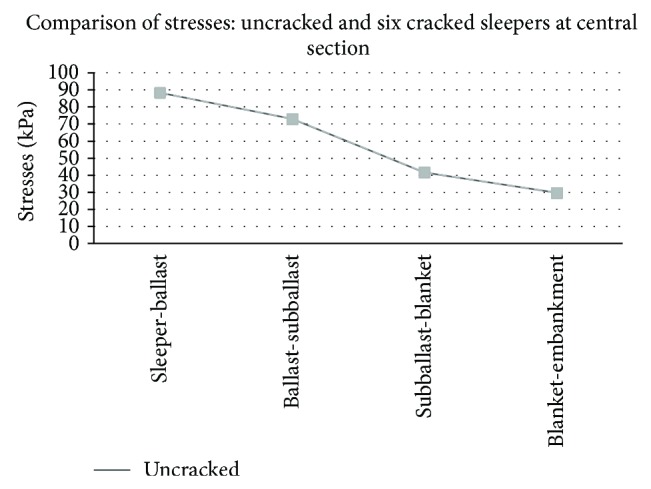
Comparison of stresses (KPa) in a track with healthy sleepers and six cracked sleepers at central section.

**Figure 8:**
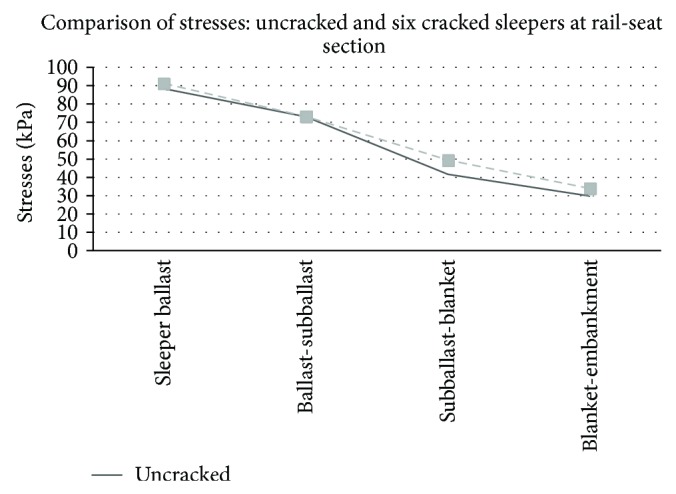
Comparison of stresses (KPa) in a track without cracks and a track with six cracked sleepers at rail-seat section.

Graphical comparison between the results obtained in the track model without cracks and the worst case (group of six cracked sleepers) may be observed in Figures 5, 6, 7, and 8.

## 5. Conclusions

The conclusions obtained after analysing the results may be also divided into the cracks located at central section and cracks located at rail-seat section.

First, regarding the models with cracked sleepers at the central section, the variation that suffered both deflections and stresses was negligible, even in the worst situation with six consecutive cracked sleepers. It was an expected conclusion since the central crack introduced a very light flexibility and was located far from the result points. Considering the sleepers renewal criteria, these cracks may be observed during the visual inspections; however, they turned out to be insignificant in this case study.

On the contrary, railway track models with different configurations of rail-seat cracks presented observable increases in both deflections and stresses. The worst situation showed a maximum increment of the deflections under the subballast layer of 16.73%. The maximum vertical deflection was registered at the rail head, with an increase of 4.7% over the reference data of the track with uncracked sleepers. In all the cases, the increase was produced in the hundredth of millimetres. With reference to the stresses, the maximum increase was produced in the contact between the subballast and the blanket with a value of 18.5%, reaching stresses of 49.4 kPa. The highest stress, which was produced in the contact between the sleeper and the ballast, had a value of 91.17 kPa (3.2% of increment).

As it may be observed, a notable increase was produced in both deflections and stresses in the track models with cracks in the rail-seat sections of some sleepers. Moreover, the studied situation corresponded to the initial effects and did not contemplate the possibility of progressive damage caused by new impact loads. This situation would accentuate the effects, contributing to the continuous damage in the sleeper and ballast. Furthermore, the real cracks produced in the track are expected to be worse than those forced in the laboratory.

The increase of the deflections produces higher deformation in granular layers which could lead to track defects. Referring to the stress increase, this includes an increase in the stress level over the ballast and thus major necessity of track maintenance.

Finally, it is also relevant to study how the vertical track stiffness varies. This parameter is defined as the quotient between the vertical load applied and the maximum deflection on rail top head. Dynamic loads, stresses, comfort, and track deterioration depend on the vertical track stiffness. Considering the results obtained, the vertical stiffness diminishes more than 3% with the appearance of the first crack at the rail-seat section and almost 5% when considering six consecutive cracked sleepers. The situation with cracked sleepers at their mid-span section does not affect the vertical stiffness. It should be noted that a decrease in the vertical track stiffness allows higher vertical deflections which could be harmful for track performance. These harmful deflections may produce notably rise of dynamic overloads and progressive increase in the stresses. However, although the stresses that arrive to the sleeper are decreased due to this diminish of the vertical stiffness, the flexibility introduced in the sleeper because of the presence of the cracks has a higher effect and the final stress transmitted in the contact sleeper-ballast, and the under layers, is higher than the case without cracks. The reason lies in the bigger deflections produced in these flexible areas, which increase the stresses. The risk of resonance phenomena due to the nonsuspended masses also rises, producing an increase in the dynamic loads, accelerating the damage process in the track, and reducing the passenger's comfort. Furthermore, the critical speed of the bending wave speed in the rail is diminished, dropping the apparent vertical stiffness until limits where the running speed may be limited and the solicitations of the wheel over the rail may be amplified. Consequently, the quality track damage may augment.

## Figures and Tables

**Table 1:** Uncracked sleeper model properties.

Mass (kg)	Length (m)	Width (m)	Height (m)	*E*_rail-seat_ (Pa)	*ν*
**336**	2.6	0.278	0.218	4.5*E*10	0.25

**Table 2:** Crack properties.

Sleeper	Crack thickness (m)	*E* (Pa)
Negative cracks at central section (at the top of the sleeper)	0.02	4.25 · 10^9^
Rail-seat positive cracks (at the bottom of the sleeper)	0.04	2.8 · 10^9^

**Table 3:** Uncracked track model calibration.

	Track model	Experimental	Error
Vertical displacement (mm)	1.237	1.244	0.51%

**Table 4:** Uncracked track model validation.

	Track model	Experimental	Error
Vertical displacement (mm)	0.483	0.473	2.2%

**Table 5:** Material properties.

Material	*E* (Pa)	*ν*	*c* (N/m^2^)	Φ_*i*_ (°)	Φ_*d*_ (°)	*ρ* (kg/m^3^)
Rail steel	2.1*E*11	0.3	—	—	—	7500
Rail pad	5.995*E*8	0.45	—	—	—	2000
Sleeper-element 1	7.04*E*10	0.25	—	—	—	2132.38
Sleeper-element 2	4.5*E*10	0.25	—	—	—	2132.38
Sleeper-element 3	3.86*E*10	0.25	—	—	—	2132.38
Sleeper-element 4	3.14*E*10	0.25	—	—	—	2132.38
Ballast	1.3*E*8	0.2	0	45	45	1900
Subballast	1.2*E*8	0.3	0	45	45	1900
Blanket	8*E*7	0.4	15000	10	10	2000
Embankment	7*E*7	0.3	15000	10	10	2000
Foundation	3*E*9	0.2	0	35	35	2000

**Table 6:** Deflections (mm) in the track model with 1 to 6 central cracks.

	*y*_rail head_	*y*_over sleeper_	*y*_under sleeper_	*y*_under ballast_	*y*_under sub ballast_	*y*_under blanket_	*y*_pad_
Uncracked	−1.238	−1.155	−1.150	−0.921	−0.672	−0.346	−0.083
Central cracks							
1	−1.238	−1.155	−1.151	−0.921	−0.671	−0.346	−0.083
2	−1.238	−1.155	−1.151	−0.921	−0.672	−0.346	−0.083
3	−1.238	−1.155	−1.151	−0.921	−0.672	−0.346	−0.083
4	−1.238	−1.155	−1.151	−0.921	−0.672	−0.346	−0.083
5	−1.238	−1.156	−1.151	−0.921	−0.672	−0.346	−0.083
6	−1.238	−1.156	−1.151	−0.921	−0.672	−0.346	−0.083

**Table 7:** Stresses (KPa) in the track model with 1 to 6 central cracks.

	*σ*_sleeper-ballast_	*σ*_ballast-sub ballast_	*σ*_sub ballast-blanket_	*σ*_blanket-embankment_
Uncracked	−88.3116	−72.9783	−41.7266	−29.8107
Central cracks				
1	−88.4412	−73.0622	−41.7243	−29.7994
2	−88.4503	−73.0677	−41.7265	−29.7992
3	−88.4527	−73.0727	−41.7285	−29.7997
4	−88.4533	−73.0743	−41.7289	−29.8004
5	−88.4560	−73.0760	−41.7295	−29.8010
6	−88.4569	−73.0767	−41.7299	−29.8013

**Table 8:** Deflections (mm) in the track model with 1 to 6 rail-seat cracks.

	*y*_rail head_	*y*_ over sleeper_	*y*_ under sleeper_	*y*_under ballast_	*y*_under sub ballast_	*y*_under blanket_	*y*_pad_
Uncracked	−1.238	−1.155	−1.150	−0.921	−0.672	−0.346	−0.083
Rail-seat cracks							
1	−1.278	−1.211	−1.195	−0.963	−0.797	−0.397	−0.067
2	−1.286	−1.218	−1.203	−0.969	−0.801	−0.398	−0.068
3	−1.293	−1.225	−1.210	−0.974	−0.805	−0.399	−0.068
4	−1.295	−1.227	−1.211	−0.975	−0.806	−0.399	−0.068
5	−1.296	−1.228	−1.212	−0.977	−0.807	−0.400	−0.068
6	−1.296	−1.228	−1.213	−0.977	−0.807	−0.400	−0.068

**Table 9:** Stresses (KPa) in the track model with 1 to 6 rail-seat cracks.

	*σ*_sleeper-ballast_	*σ*_ ballast-sub ballast_	*σ*_sub ballast-blanket_	*σ*_ blanket-embankment_
Uncracked	−88.3116	−72.9783	−41.7266	−29.8107
Rail-seat cracks				
1	−89.6458	−72.0105	−48.8820	−33.7358
2	−90.6207	−72.6040	−49.1332	−33.7492
3	−91.2225	−73.0537	−49.3672	−33.8167
4	−91.1491	−73.0612	−49.4115	−33.8595
5	−91.1764	−73.1009	−49.4411	−33.8931
6	−91.1765	−73.1029	−49.4426	−33.8970

## Notations

$E_{\text{model}}$: Modulus of elasticity modeled
$E_{\text{real}}$: Modulus of elasticity real
$I_{\text{model}}$: Inertia modulus modeled
$I_{\text{real}}$: Inertia modulus real
[M]: Mass matrix
$\left\{\ddot{u}\right\}$: Acceleration vector
[K]: Stiffness matrix
[u]: Displacement vector
E: Young’s modulus
k: Vertical stiffness’ rail pad
h: Rail pad thickness
s: Rail pad contact surface
$Q_{d}$: Dynamic load
$Q_{e}$: Vehicle static load
t: Statistical security coefficient
$\overset{-}{s}$: Factor of the quality conditions of the track
v: Speed of vehicle.
